# Associations of *HLA* Polymorphisms with Chronic Kidney Disease in Japanese Rheumatoid Arthritis Patients

**DOI:** 10.3390/genes14071470

**Published:** 2023-07-19

**Authors:** Takashi Higuchi, Shomi Oka, Hiroshi Furukawa, Kota Shimada, Atsushi Hashimoto, Akiko Komiya, Toshihiro Matsui, Naoshi Fukui, Shigeto Tohma

**Affiliations:** 1 Department of Rheumatology, National Hospital Organization Tokyo National Hospital, 3-1-1 Takeoka, Kiyose 204-8585, Japan; takashi.qef@ac.auone-net.jp (T.H.); oka-tkb@umin.org (S.O.); touma.shigeto.jy@mail.hosp.go.jp (S.T.); 2 Department of Nephrology, Ushiku Aiwa General Hospital, 896 Shishiko-cho, Ushiku 300-1296, Japan; 3 Clinical Research Center for Allergy and Rheumatology, National Hospital Organization Sagamihara National Hospital, 18-1 Sakuradai, Minami-ku, Sagamihara 252-0392, Japan; komiya.akiko.zy@mail.hosp.go.jp (A.K.); ninja02matsui@gmail.com (T.M.); n-fukui@idaten.c.u-tokyo.ac.jp (N.F.); 4 Department of Rheumatology, National Hospital Organization Sagamihara National Hospital, 18-1 Sakuradai, Minami-ku, Sagamihara 252-0392, Japan; kouta_shimada@tmhp.jp (K.S.); hako@happy.email.ne.jp (A.H.); 5 Department of Rheumatic Diseases, Tokyo Metropolitan Tama Medical Center, 2-8-29 Musashi-dai, Fuchu 183-8524, Japan; 6 Department of Internal Medicine, Sagami Seikyou Hospital, 6-2-11 Sagamiohno, Minami-ku, Sagamihara 252-0303, Japan; 7 Department of Clinical Laboratory, National Hospital Organization Sagamihara National Hospital, 18-1 Sakuradai, Minami-ku, Sagamihara 252-0392, Japan; 8 Department of Life Sciences, Graduate School of Arts and Sciences, The University of Tokyo, 3-8-1 Komaba, Meguro-ku, Tokyo 153-8902, Japan

**Keywords:** *HLA*, chronic kidney disease, rheumatoid arthritis

## Abstract

Objectives: The prevalence of chronic kidney disease (CKD) was reported to be higher in rheumatoid arthritis (RA) patients than in normal healthy individuals. Human leukocyte antigen (*HLA*) was associated with RA or CKD. Few studies on the association of *HLA* with CKD in RA have been reported. Here, we investigated the association of *HLA* polymorphisms with CKD in Japanese RA patients. Methods: *HLA-DRB1* genotyping was conducted in 351 Japanese RA patients with CKD (estimated glomerular filtration rate [eGFR] lower than 60 [mL/min/1.73 m^2^]) and 959 without CKD (eGFR equal to or higher than 60 [mL/min/1.73 m^2^]). Associations of allele carrier frequencies of *DRB1* with CKD were examined in the RA patients. Results: There was an association of DRB1*13:02 with CKD in RA, but this did not achieve statistical significance (*p* = 0.0265, odds ratio [OR] 1.70, *p*c = 0.7412, 95% confidence interval [CI] 1.09–2.64). The DR6 serological group was associated with CKD in RA (*p* = 0.0008, OR 1.65, 95% CI 1.24–2.20). A gene-dosage effect of DR6 was not detected. Logistic regression analysis showed that the association of DR6 with CKD in RA was independent of clinical characteristics. Conclusions: The present study first revealed the independent predisposing association of DR6 with CKD in Japanese RA patients, although DR6 is known to be protective against RA. Our data suggest direct or indirect roles of *HLA* for the development of CKD in RA, but the mechanisms are not clear.

## 1. Introduction

Rheumatoid arthritis (RA) is a systemic autoimmune disease characterized by the destruction of synovial joints. RA is commonly complicated with chronic kidney disease (CKD) [1,2,3,4], and the prevalence of CKD is higher in RA patients than that in the general population [5,6,7]. Several factors, including age and complications of hypertension, diabetes mellitus, secondary amyloidosis, rheumatoid vasculitis, drug-induced membranous nephropathy, and interstitial nephritis, are involved in the pathogenesis of CKD in RA [8,9]. Inflammation was reported to be a risk factor for the development of CKD in RA [10], and treatment with biological agents decreased the risk [11]. However, the cause of CKD is generally difficult to determine in most RA patients.

Human leukocyte antigen (*HLA*)-*DRB1* is the most important genetic risk factor for RA. Many studies have reported the association of *HLA* polymorphisms with susceptibility to RA [12,13,14] or CKD [15,16]. Different DRB1 alleles associated with RA are dependent upon various ethnicities: DRB1*04:01 is associated with European RA [12], and DRB1*04:05 is associated with Japanese RA [13]. Amino acid residues at positions 70–74 in the DRβ chain were conserved as risk *DRB1* alleles for RA, and these are called shared epitope (SE) alleles [12]. A gene-dosage effect was observed for the associations of *DRB1* alleles with RA: homozygosity for risk alleles conferred a higher odds ratio (OR) than heterozygosity. Different *HLA* alleles have been associated with CKD in different studies [16], which might be explained by different underlying diseases or different ethnicities. However, few studies have examined the association of *HLA* with CKD in RA patients to date. Here, we evaluated the association of *HLA* polymorphisms with CKD in Japanese RA patients.

## 2. Materials and Methods

### 2.1. Patients and Controls

A total of 1310 RA patients were recruited at Sagamihara National Hospital and Tokyo National Hospital. All RA patients fulfilled the American College of Rheumatology Criteria for RA or Rheumatoid Arthritis Classification Criteria [17,18]. A total of 413 healthy individuals were recruited from Sagamihara National Hospital, Teikyo University, Kanazawa University, or by the Pharma SNP Consortium (Tokyo, Japan) [19,20]. Patients and healthy individuals were native Japanese living in Japan. The estimated glomerular filtration rate (eGFR) was calculated using the equation of the Japanese Society of Nephrology [21]: eGFR (mL/min/1.73 m^2^) = 194 × serum creatinine^−1.094^ × age^−0.287^ × 0.739 (if female). RA patients with an eGFR lower than 60 (mL/min/1.73 m^2^) were defined as CKD(+)RA. RA patients with an eGFR equal to or higher than 60 (mL/min/1.73 m^2^) were defined as CKD(−)RA.

The protocol of this study was reviewed and approved by the Research Ethics Committee of Tokyo National Hospital (190010) and Sagamihara National Hospital. Written informed consent was obtained from each participant. This study was conducted in accordance with the principles expressed in the Declaration of Helsinki.

### 2.2. Genotyping

The genotyping of *HLA-DRB1* was performed by polymerase chain reaction with reverse sequence-specific oligonucleotide probes (WAKFlow HLA typing kits, Wakunaga, Akitakata, Japan), using the Bio-Plex system (Bio-Rad, Hercules, CA, USA). Genotyping results for some RA patients and healthy controls were reported in previous studies [13,22].

### 2.3. Statistical Analysis

Differences in the clinical characteristics between RA patients were analyzed by Fisher’s exact test using 2 × 2 contingency tables or the Student’s *t*-test. Associations of *DRB1* allele carrier frequencies or genotype frequencies, or amino acid residue carrier frequencies were tested by Fisher’s exact test using 2 × 2 contingency tables. Deviation from Hardy–Weinberg equilibrium was detected by Genepop (http://genepop.curtin.edu.au/ (accessed on 13 January 2023)) [23]. Multiple logistic regression analysis under the additive model was used to examine whether *DRB1* alleles were independently associated with CKD in RA patients. The corrected *p* (*p*c) values were generated by multiplying the *p*-values by the number of alleles or amino acid residues tested. Principal component analysis (PCA) was performed to discriminate between CKD(+)RA, CKD(−)RA, and healthy control groups based on the allele frequencies of *DRB1*.

## 3. Results

### 3.1. Clinical Characteristics of RA Patients

The clinical characteristics of RA patients are shown in Table 1. Of all RA patients, 351 are defined as CKD(+)RA and 959 as CKD(−)RA. CKD(+)RA patients are older than CKD(−)RA patients. The Steinbrocker class [24], body mass index, erythrocyte sedimentation rate, and disease activity score 28 (DAS28) are higher in CKD(+)RA patients than in CKD(−)RA patients. 

### 3.2. Association of HLA with CKD in RA Patients

*DRB1* genotyping was conducted to compare allele carrier frequencies between RA patients with or without CKD (Table 2). Deviation from Hardy–Weinberg equilibrium is found in the CKD(−)RA group (*p* = 0.0140) but not in the CKD(+)RA group (*p* = 0.6059). There is an association of DRB1*13:02 with CKD in RA patients, but this does not achieve statistical significance (*p* = 0.0265, OR 1.70, *p*c = 0.7412, 95% confidence interval [CI] 1.09–2.64). The DR6 serological group is associated with CKD in RA patients (*p* = 0.0008, OR 1.65, 95% CI 1.24–2.20), indicating that DR6 is predisposing to CKD in RA. *DRB1* genotype frequencies in the CKD(+)RA patients are compared with those of the CKD(−)RA patients to clarify the presence or absence of gene-dosage effects (Table 3). Homozygosity for DR6 does not have a higher risk for CKD compared to heterozygosity (DR6/not DR6: *p* = 0.0009, OR 1.66, 95%CI 1.24–2.23, DR6/DR6: *p* = 0.7558, OR 1.22, 95%CI 0.37–3.98), indicating the absence of the gene-dosage effect. The allele carrier frequency of *DRB1* was also compared with those of healthy controls to confirm the protective role of DR6 against RA in CKD(+)RA group (Supplementary Table S1). The allele carrier frequency of DR6 is lower than that in healthy controls (*p* = 0.0481, OR 0.73, 95%CI 0.53–0.99), indicating that DR6 is still protective against RA in CKD(+)RA group. Thus, DR6 is associated with CKD in RA patients, although a gene-dosage effect is not observed.

### 3.3. PCA of the CKD(+)RA, CKD(−)RA, and Healthy Control Groups

PCA was performed to discriminate between the CKD(+)RA, CKD(−)RA, and healthy control groups based on the allele frequencies of *DRB1* (Supplementary Figure S1). The results of PCA component 1 are related to the division of RA and healthy controls, although the results of PCA component 2 seem to explain the presence of CKD. Thus, CKD(+)RA group is different from CKD(−)RA or the healthy control groups. 

### 3.4. Associations of Amino Acid Residues in the DRβ Chain of RA Patients with CKD

**Figure 1:**
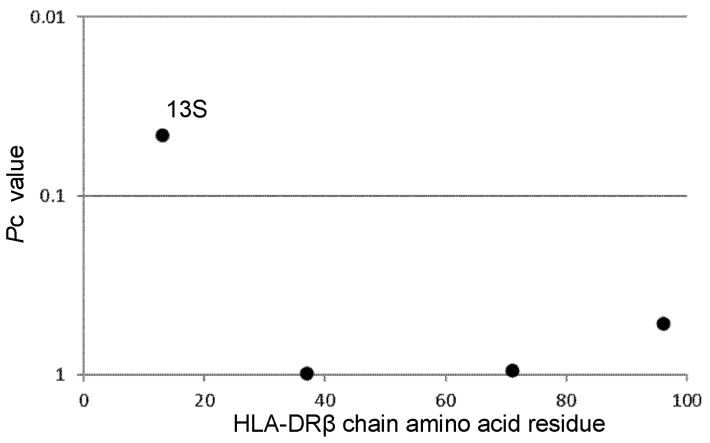
Associations of amino acid residues in the DRβ chain with CKD in RA patients. The significance of the associations was established by Fisher’s exact test, using 2 × 2 contingency tables. Corrected *p* (pc) values were generated by multiplying *p*-values by the number of analyzed amino acid residues. Predisposing associations are indicated by filled circles. RA: rheumatoid arthritis; CKD: chronic kidney disease.

Associations of amino acid residues in the HLA-DRβ chain with CKD in RA patients were analyzed to reveal the effects of amino acid residues on the predisposition to or protection against CKD (Figure 1). Serine at position 13 (13S, *p* = 0.0013, OR 1.59, *p*c = 0.0456, 95% CI 1.21–2.11) is associated with CKD in RA patients. Thus, one amino acid residue is associated with CKD, probably through the presentation of antigens.

### 3.5. Logistic Regression Analysis of DR6 and Clinical Characteristics of CKD

Because older RA patients are prone to CKD, multiple logistic regression analyses of DR6 and the clinical characteristics of patients were performed to exclude the influence of clinical characteristics on the pathogenesis of CKD (Table 4). The association of DR6 remains significant (*p*_adjusted_ = 0.0196, OR_adjusted_ 1.43, 95% CI 1.06–1.94) when conditioned on the clinical characteristics, suggesting the independent association of DR6 with CKD in RA patients. Age, Steinbrocker class, and body mass index remain associated with CKD when conditioned on other factors. Thus, DR6 is independently associated with CKD in RA patients.

## 4. Discussion

The present study revealed the independent association of DR6 with CKD in Japanese RA patients, although DR6 is protective against RA and other autoimmune diseases [13,22,25]. Our data suggest direct or indirect roles of *HLA* in the development of CKD in RA. Bucillamine, one of the disease-modifying anti-rheumatic drugs, occasionally causes drug-induced membranous nephropathy in RA patients. Bucillamine-induced membranous nephropathy in RA was associated with *HLA* [26]. Analogically, it is possible that DR6 is associated with drug-induced membranous nephropathy in RA patients. It could be suggested that other common genetic or environmental factors than *HLA* might contribute to the pathogenesis of both RA and CKD in RA patients with DR6. Single nucleotide polymorphisms, rare variants, structural variants outside of the *HLA* region, or smoking would contribute to the pathogenesis. Otherwise, drugs used in the treatment for RA could cause CKD in RA patients, and it would influence the results of this study.

The associations of *HLA* with RA have also been reported by many studies [12,13,14]. The association of SE with RA was reported and DRB1*01:01, DRB1*04:01, DRB1*04:04, DRB1*04:05, DRB1*04:10, DRB1*10:01, DRB1*14:02, and DRB1*14:06 were designated as SE alleles [12]. In this study, the allele carrier frequencies of SE alleles were not increased in CKD(+)RA patients compared to CKD(−)RA patients but were increased when compared with healthy controls. The protective association of DR6 with RA was reported previously [13], and DR6 includes the DRB1*13 and DRB1*14 alleles. The allele carrier frequencies of DR6 were increased in CKD(+)RA patients compared with CKD(−)RA patients but were decreased compared with healthy controls. These data suggested that DR6 alleles were still protective against RA, though they were predisposing to CKD. Thus, the *DRB1* distribution pattern in CKD(+)RA patients is different from that in CKD(−)RA patients or healthy controls.

The current study detected an association of amino acid residue 13S in the DRβ chain with CKD in RA patients. The 13S residue is encoded by DRB1*13:02, DRB1*14:06, and DRB1*14:54; thus, the association of amino acid residue 13S reflects the predisposing effects of DRB1*13:02, DRB1*14:06, and DRB1*14:54 alleles for the development of CKD.

Conditioned logistic regression analyses suggested that DR6 independently contributed to the susceptibility of RA patients to CKD. Age, Steinbrocker class, and body mass index were also associated with CKD in RA patients. Age was an essential factor in the estimation of physiological renal function and is included in the equation for eGFR [21]. The increase in Steinbrocker’s class might have been a result of CKD in RA patients. Obesity found in CKD(+)RA patients might be explained by obesity-related chronic kidney disease associated with hypertension, diabetes mellitus, or the impaired production of adipokine or proinflammatory cytokines. However, other confounding factors could not be eliminated. Renal function in RA patients might also be affected by longstanding inflammation or the side effects of drugs [7,10,11]. DR6 was revealed to be a risk factor for CKD in RA patients and, therefore, might be useful for the prevention of CKD in RA patients with DR6: newly diagnosed RA patients should be genotyped, and RA patients with DR6 should be under the strict management of blood pressure or blood glucose levels. Because some drugs are excreted in the urine, the selection of therapeutic strategies for CKD(+)RA is tightly restricted. This prevention strategy for CKD in RA patients might help for better controls for RA.

The present study had some limitations. The sample size of this study was modest and only included those in the Japanese population. The distribution patterns of *DRB1* alleles are different in other European or Asian ethnic populations. Although an association of DR6 was found in this study, other culprit genes in linkage disequilibrium with *DRB1* loci might be involved in the pathogenesis of CKD. The associations of other *HLA* loci with CKD in RA patients should be investigated. Larger multiethnic studies of the total *HLA* region should be performed to confirm the associations of DR6 with CKD in RA patients. Data related to albuminuria were not available, and this might affect the diagnosis of CKD in RA patients and the results obtained in the present study. In conclusion, the present study revealed the independent association of DR6 with CKD in Japanese RA patients.

## Figures and Tables

**Table 1:** Characteristics of RA patients.

	CKD(+)RA	CKD(−)RA	
Number of patients	351	959	
Age, years (SD)	73.5 (8.6)	64.2 (12.4)	* 2.99 × 10^−47^
Male, n (%)	73 (20.8)	161 (16.8)	0.1033
Disease duration, years (SD)	17.7 (12.5)	16.3 (11.3)	* 0.0632
Steinbrocker stage III and IV, n (%)	156 (45.3)	418 (44.1)	0.7043
Steinbrocker class 3 and 4, n (%)	61 (17.8)	96 (10.1)	0.0003
Body mass index, kg/m^2^ (SD)	22.3 (3.9)	21.7 (3.5)	* 0.0171
Rheumatoid factor positive, n (%)	287 (82.0)	766 (80.5)	0.5782
Anti-citrullinated peptide antibody positive, n (%)	303 (86.6)	823 (86.7)	0.9269
Serum creatinine, mg/dL (SD)	0.96 (0.25)	0.62 (0.12)	* 2.71 × 10^−82^
CRP, mg/L (SD)	4.6 (7.5)	5.1 (11.8)	* 0.3548
ESR, mm/h (SD)	30.2 (21.0)	26.2 (21.1)	* 0.0035
DAS28	3.0 (1.0)	2.8 (1.1)	* 0.0125
DAS28-CRP	2.2 (0.9)	2.2 (0.9)	* 0.3548

Number or average values of each group are shown. Standard deviations or percentages are shown in parentheses. Differences were tested by Fisher’s exact test using 2 × 2 contingency tables or the Student’s *t*-test. * The Student’s *t*-test was used. RA: rheumatoid arthritis; CKD: chronic kidney disease; CRP: C-reactive protein; ESR: erythrocyte sedimentation rate; DAS28: disease activity score 28.

**Table 2:** *DRB1* allele carrier frequency in RA patients with or without CKD.

	CKD(+)RA (n = 351)	CKD(−)RA (n = 959)	*p*	OR	*p*c	95%CI
DRB1*01:01	54 (15.4)	136 (14.2)	0.5955	1.10	NS	(0.78–1.55)
DRB1*03:01	1 (0.3)	0 (0.0)	0.2679	8.21	NS	(0.33–202.08)
DRB1*04:01	19 (5.4)	75 (7.8)	0.1478	0.67	NS	(0.40–1.13)
DRB1*04:03	10 (2.8)	28 (2.9)	1.0000	0.98	NS	(0.47–2.03)
DRB1*04:04	3 (0.9)	5 (0.5)	0.4483	1.64	NS	(0.39–6.92)
DRB1*04:05	162 (46.2)	487 (50.8)	0.1513	0.83	NS	(0.65–1.06)
DRB1*04:06	11 (3.1)	45 (4.7)	0.2799	0.66	NS	(0.34–1.29)
DRB1*04:07	3 (0.9)	4 (0.4)	0.3934	2.06	NS	(0.46–9.24)
DRB1*04:10	16 (4.6)	39 (4.1)	0.7557	1.13	NS	(0.62–2.04)
DRB1*07:01	2 (0.6)	5 (0.5)	1.0000	1.09	NS	(0.21–5.66)
DRB1*08:02	18 (5.1)	29 (3.0)	0.0917	1.73	NS	(0.95–3.16)
DRB1*08:03	26 (7.4)	81 (8.4)	0.6487	0.87	NS	(0.55–1.37)
DRB1*09:01	102 (29.1)	252 (26.3)	0.3258	1.15	NS	(0.88–1.51)
DRB1*10:01	5 (1.4)	18 (1.9)	0.8123	0.76	NS	(0.28–2.05)
DRB1*11:01	10 (2.8)	30 (3.1)	0.8584	0.91	NS	(0.44–1.88)
DRB1*12:01	21 (6.0)	70 (7.3)	0.4625	0.81	NS	(0.49–1.34)
DRB1*12:02	9 (2.6)	27 (2.8)	1.0000	0.91	NS	(0.42–1.95)
DRB1*13:01	0 (0.0)	2 (0.2)	1.0000	0.54	NS	(0.03–11.38)
DRB1*13:02	34 (9.7)	57 (5.9)	0.0265	1.70	0.7412	(1.09–2.64)
DRB1*14:02	1 (0.3)	2 (0.2)	1.0000	1.37	NS	(0.12–15.12)
DRB1*14:03	6 (1.7)	21 (2.2)	0.6672	0.78	NS	(0.31–1.94)
DRB1*14:05	7 (2.0)	18 (1.9)	0.8237	1.06	NS	(0.44–2.57)
DRB1*14:06	19 (5.4)	30 (3.1)	0.0691	1.77	NS	(0.98–3.19)
DRB1*14:07	1 (0.3)	1 (0.1)	0.4642	2.74	NS	(0.17–43.88)
DRB1*14:54	27 (7.7)	46 (4.8)	0.0559	1.65	NS	(1.01–2.70)
DRB1*15:01	37 (10.5)	116 (12.1)	0.4967	0.86	NS	(0.58–1.27)
DRB1*15:02	56 (16.0)	163 (17.0)	0.6769	0.93	NS	(0.67–1.29)
DRB1*16:02	5 (1.4)	14 (1.5)	1.0000	0.98	NS	(0.35–2.73)
DR6 (DRB1*13, *14)	93 (26.5)	172 (17.9)	0.0008	1.65		(1.24–2.20)

Allele carrier frequencies are shown in parentheses (%). Associations were tested by Fisher’s exact test using 2 × 2 contingency tables. RA: rheumatoid arthritis; CKD: chronic kidney disease; OR: odds ratio; CI: confidence interval; *p*c: corrected *p*.

**Table 3:** *HLA-DRB1* genotype frequency in RA patients with or without CKD.

	CKD(+)RA (n = 351)	CKD(−)RA (n = 959)	*p*	OR	95%CI
DR6/not DR6	89 (25.4)	163 (17.0)	0.0009	1.66	(1.24–2.23)
DR6/DR6	4 (1.1)	9 (0.9)	0.7558	1.22	(0.37–3.98)

Genotype frequencies are shown in parentheses (%). Associations were tested by Fisher’s exact test, using 2 × 2 contingency tables. RA: rheumatoid arthritis; CKD: chronic kidney disease; OR: odds ratio; CI: confidence interval.

**Table 4:** Multiple logistic regression analysis of DR6 and clinical manifestations for CKD in RA patients.

	Unconditioned			Conditioned on Other Clinical Manifestations		
Clinical Manifestations	OR	95%CI	*p*	OR_adjusted_	95%CI	*p*_adjusted_
Age	1.10	(1.08–1.11)	2.95 × 10^−30^	1.10	(1.08–1.11)	4.68 × 10^−25^
Male	1.30	(0.96–1.77)	0.0940	1.10	(0.77–1.57)	0.5978
Disease duration	1.01	(1.00–1.02)	0.0517	0.99	(0.98–1.01)	0.3772
Steinbrocker class	1.32	(1.10–1.59)	0.0024	1.24	(1.00–1.52)	0.0475
Body mass index	1.04	(1.01–1.08)	0.0126	1.05	(1.01–1.09)	0.0176
ESR	1.01	(1.00–1.01)	0.0040	1.00	(0.99–1.01)	0.7025
DR6	1.55	(1.19–2.03)	0.0013	1.43	(1.06–1.94)	0.0196

*p*, OR, 95%CI, *p*_adjusted_, and OR_adjusted_ were calculated by logistic regression analysis of RA patients. RA: rheumatoid arthritis; CKD: chronic kidney disease; OR: odds ratio; CI: confidence interval.

## Data Availability

Data supporting the findings of this study are presented in the paper and the supplementary file. Other data are available from the authors upon reasonable request. However, the clinical information and genotype data of each participant are not available under the conditions of informed consent mandated by the Act on the Protection of Personal Information.

## Supplementary Materials

The following supporting information can be downloaded at: https://www.mdpi.com/article/10.3390/genes14071470/s1, Figure S1: Results of principal component analyses of CKD(+)RA, CKD(−)RA, and healthy control groups based on allele frequencies of *DRB1*; Table S1: *DRB1* allele carrier frequency in RA patients with CKD and controls.
