# Supplementary material for: Associations of HLA Polymorphisms with Chronic Kidney Disease in Japanese Rheumatoid Arthritis Patients
Source: Genes (Basel). 2023 Jul 19;14(7):1470. doi: 10.3390/genes14071470 (PMC10379419; doi:10.3390/genes14071470)
Supplement: Supplementary file 1 [file genes-14-01470-s001.zip › HLACKDRAFigure#5-S1.pdf]

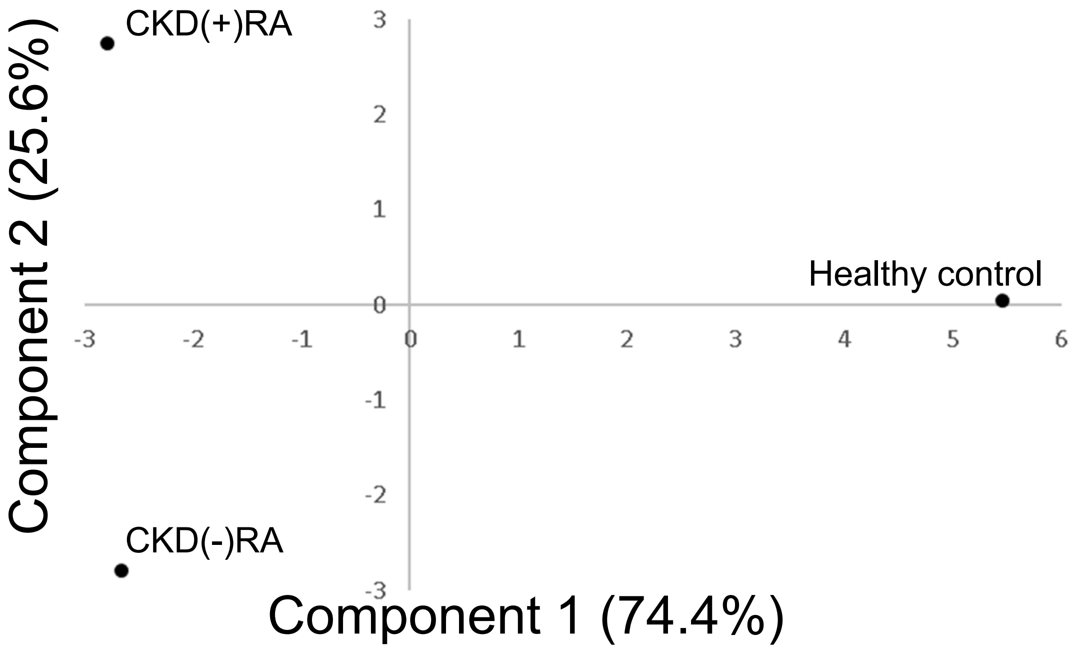

Supplementary Figure S1. Results of principal component analyses of CKD(+)RA, CKD(-)RA, and healthy control groups based on allele frequencies of *DRB1*. Scores plots for principal component analyses of CKD(+)RA, CKD(-)RA, and healthy controls. RA: rheumatoid arthritis, CKD: chronic kidney disease.
