# Supplementary material for: Associations of HLA Polymorphisms with Chronic Kidney Disease in Japanese Rheumatoid Arthritis Patients
Source: Genes (Basel). 2023 Jul 19;14(7):1470. doi: 10.3390/genes14071470 (PMC10379419; doi:10.3390/genes14071470)
Supplement: Supplementary file 1 [file genes-14-01470-s001.zip › HLACKDRATable#3-S1.pdf]

Supplemenatry Table S1. *DRB1* allele carrier frequency in RA patients with CKD and controls.

| | CKD(+)RA (n=351) | Healthy control (n=413) | *P* | OR | *P*c | 95%CI |
|---|---|---|---|---|---|---|
| *DRB1*01:01* | 54 (15.4) | 41 (9.9) | 0.0275 | 1.65 | 0.8247 | (1.07-2.55) |
| *DRB1*03:01* | 1 (0.3) | 2 (0.5) | 1.0000 | 0.59 | NS | (0.05-6.50) |
| *DRB1*04:01* | 19 (5.4) | 7 (1.7) | 0.0080 | 3.32 | 0.2397 | (1.38-7.99) |
| *DRB1*04:03* | 10 (2.8) | 19 (4.6) | 0.2554 | 0.61 | NS | (0.28-1.33) |
| *DRB1*04:04* | 3 (0.9) | 0 (0.0) | 0.0965 | 8.31 | NS | (0.43-161.35) |
| *DRB1*04:05* | 162 (46.2) | 87 (21.1) | $2.04X10^{-13}$ | 3.21 | $6.11X10^{-12}$ | (2.34-4.41) |
| *DRB1*04:06* | 11 (3.1) | 34 (8.2) | 0.0031 | 0.36 | 0.0926 | (0.18-0.72) |
| *DRB1*04:07* | 3 (0.9) | 3 (0.7) | 1.0000 | 1.18 | NS | (0.24-5.87) |
| *DRB1*04:10* | 16 (4.6) | 14 (3.4) | 0.4572 | 1.36 | NS | (0.65-2.83) |
| *DRB1*07:01* | 2 (0.6) | 3 (0.7) | 1.0000 | 0.78 | NS | (0.13-4.71) |
| *DRB1*08:02* | 18 (5.1) | 38 (9.2) | 0.0364 | 0.53 | NS | (0.30-0.95) |
| *DRB1*08:03* | 26 (7.4) | 61 (14.8) | 0.0013 | 0.46 | 0.0405 | (0.28-0.75) |
| *DRB1*08:09* | 0 (0.0) | 1 (0.2) | 1.0000 | 0.39 | NS | (0.02-9.63) |
| *DRB1*09:01* | 102 (29.1) | 105 (25.4) | 0.2884 | 1.20 | NS | (0.87-1.65) |
| *DRB1*10:01* | 5 (1.4) | 2 (0.5) | 0.2571 | 2.97 | NS | (0.57-15.40) |
| *DRB1*11:01* | 10 (2.8) | 21 (5.1) | 0.1419 | 0.55 | NS | (0.25-1.18) |
| *DRB1*12:01* | 21 (6.0) | 29 (7.0) | 0.6601 | 0.84 | NS | (0.47-1.51) |
| *DRB1*12:02* | 9 (2.6) | 10 (2.4) | 1.0000 | 1.06 | NS | (0.43-2.64) |
| *DRB1*13:01* | 0 (0.0) | 5 (1.2) | 0.0658 | 0.11 | NS | (0.01-1.92) |
| *DRB1*13:02* | 34 (9.7) | 57 (13.8) | 0.0927 | 0.67 | NS | (0.43-1.05) |
| *DRB1*14:02* | 1 (0.3) | 0 (0.0) | 0.4594 | 3.54 | NS | (0.14-87.16) |
| *DRB1*14:03* | 6 (1.7) | 21 (5.1) | 0.0168 | 0.32 | 0.5028 | (0.13-0.81) |
| *DRB1*14:04* | 0 (0.0) | 1 (0.2) | 1.0000 | 0.39 | NS | (0.02-9.63) |
| *DRB1*14:05* | 7 (2.0) | 14 (3.4) | 0.2732 | 0.58 | NS | (0.23-1.45) |
| *DRB1*14:06* | 19 (5.4) | 16 (3.9) | 0.3857 | 1.42 | NS | (0.72-2.81) |
| *DRB1*14:07* | 1 (0.3) | 1 (0.2) | 1.0000 | 1.18 | NS | (0.07-18.89) |
| *DRB1*14:54* | 27 (7.7) | 28 (6.8) | 0.6745 | 1.15 | NS | (0.66-1.98) |
| *DRB1*15:01* | 37 (10.5) | 68 (16.5) | 0.0202 | 0.60 | 0.6060 | (0.39-0.92) |
| *DRB1*15:02* | 56 (16.0) | 89 (21.5) | 0.0522 | 0.69 | NS | (0.48-1.00) |
| *DRB1*16:02* | 5 (1.4) | 5 (1.2) | 1.0000 | 1.18 | NS | (0.34-4.11) |
| DR6(*DRB1*13, *14*) | 93 (26.5) | 137 (33.2) | 0.0481 | 0.73 | | (0.53-0.99) |

Allele carrier frequencies are shown in parentheses (%). Association was tested by Fisher's exact test using 2X2 contingency tables. RA: rheumatoid arthritis, CKD: chronic kidney disease, OR: odds ratio, CI: confidence interval, *P*c: corrected *P*.
